# Identification of cancer prognosis-associated functional modules using differential co-expression networks

**DOI:** 10.18632/oncotarget.22878

**Published:** 2017-12-04

**Authors:** Wenshuai Yu, Shengjie Zhao, Yongcui Wang, Brian Nlong Zhao, Weiling Zhao, Xiaobo Zhou

**Affiliations:** ^1^ Key Laboratory of Embedded System and Service Computing, College of Electronics and Information Engineering, The Ministry of Education, Tongji University, Shanghai, China; ^2^ College of Software Engineering, Tongji University, Shanghai, China; ^3^ Key Laboratory of Adaptation and Evolution of Plateau Biota, Northwest Institute of Plateau Biology, Chinese Academy of Sciences, Xining, China; ^4^ Shanghai High School International Division, Shanghai, China; ^5^ Department of Radiology and Comprehensive Cancer Center, Wake Forest University School of Medicine, Winston Salem, NC, USA; ^6^ College of Electronics and Information Engineering, Tongji University, Shanghai, China; ^7^ Center for Big Data Sciences and Network Security, Tongji University, Shanghai, China; ^8^ Center for Bioinformatics and System Biology, Wake Forest University School of Medicine, Winston Salem, NC, USA

**Keywords:** co-expression network, prognosis, HO-GSVD, gene module, cancer

## Abstract

The rapid accumulation of cancer-related data owing to high-throughput technologies has provided unprecedented choices to understand the progression of cancer and discover functional networks in multiple cancers. Establishment of co-expression networks will help us to discover the systemic properties of carcinogenesis features and regulatory mechanisms of multiple cancers. Here, we proposed a computational workflow to identify differentially co-expressed gene modules across 8 cancer types by using combined gene differential expression analysis methods and a higher-order generalized singular value decomposition. Four co-expression modules were identified; and oncogenes and tumor suppressors were significantly enriched in these modules. Functional enrichment analysis demonstrated the significantly enriched pathways in these modules, including ECM-receptor interaction, focal adhesion and PI3K-Akt signaling pathway. The top-ranked miRNAs (mir-199, mir-29, mir-200) and transcription factors (*FOXO4*, *E2A*, *NFAT*, and *MAZ*) were identified, which play an important role in deregulating cellular energetics; and regulating angiogenesis and cancer immune system. The clinical significance of the co-expressed gene clusters was assessed by evaluating their predictability of cancer patients’ survival. The predictive power of different clusters and subclusters was demonstrated. Our results will be valuable in cancer-related gene function annotation and for the evaluation of cancer patients’ prognosis.

## INTRODUCTION

The rapid accumulation of cancer-related data owing to high-throughput technologies has provided unprecedented choices to understand the progression of cancer and discover functional networks in multiple cancers. The vast majority of cancer-related studies have focused on a single cancer, but always ignored the common traits across different cancer types. Different cancers usually share common hallmarks, such as evading growth suppressors, resisting cell death and inducing angiogenesis. Moreover, the methods based on biological networks including gene co-expression networks, metabolic networks, protein-protein interaction networks and genetic regulatory networks can infer regulatory mechanisms related to biological processes. The network-based methods to search biological processes related to cancer hallmarks will help us in identifying the characterizations of tumor biology. Few studies focus on genome-scale networks across different cancer types. Thus, the network analysis may help us to unveil common traits involved in multiple cancers.

The majority of the network-based methods are performed to identify distinct patterns within one cancer [1, 2]. Compared with only analyzing one cancer, several methods have been used to identify common patterns shared by two or more cancers. Zhang et al. used a network mining algorithm to build tightly connected gene co-expression networks from the microarray datasets spanning 33 cancer types. Their results indicate that the commonly recognized characteristics of cancers are supported by highly coordinated transcriptomic activities [3]. Yang et al. did the weighted correlation network analysis (WGCNA) to highlight common properties of prognostic genes in four cancer types [4]. Li et al. described a network method to analyze the driver mutation by integrating both cancer genomes and transcriptomes and identified a significant correlation of genotype to phenotype in six solid tumors [5].

Several computationally efficient methods have been developed for construction of the networks, such as Generalized Singular Value Decomposition (GSVD) [6] and higher-order GSVD (HO-GSVD) [7]. GSVD is used for identifying common structures across two conditions [6]. The analysis based on HO-GSVD can extract common gene modules in two or more conditions [7–9]. Ponnapalli et al. first proposed HO-GSVD to analyze common structures shared by multiple datasets [7]. Xiao et al. developed a new HO-GSVD method for analyzing common and tissue-specific modules from seven rat tissues and four human brain regions [8]. Wang et al. applied a simple mathematical framework of HO-GSVD for analysis of multiple tissues [9]. These studies indicate that HO-GSVD is a valuable method in common gene pattern discovery among different tissues. Motivated by this approach, we applied HO-GSVD to pan-cancer analysis in this study. To our best of knowledge, this approach has not been used for pan-cancer research.

Differential gene expression analysis has been widely used for identifying differentially expressed genes between conditions. The commonly used methods include edgeR [10], limma [11], SAMseq [12] and DESeq [13]. Most studies only use one method to analyze gene expression patterns. Soneson et al. compared the commonly used methods for differential expression analysis and found that no single method is optimal under all circumstances and the method of choice in a particular situation depends on the experimental conditions [14].

**Figure 1:**
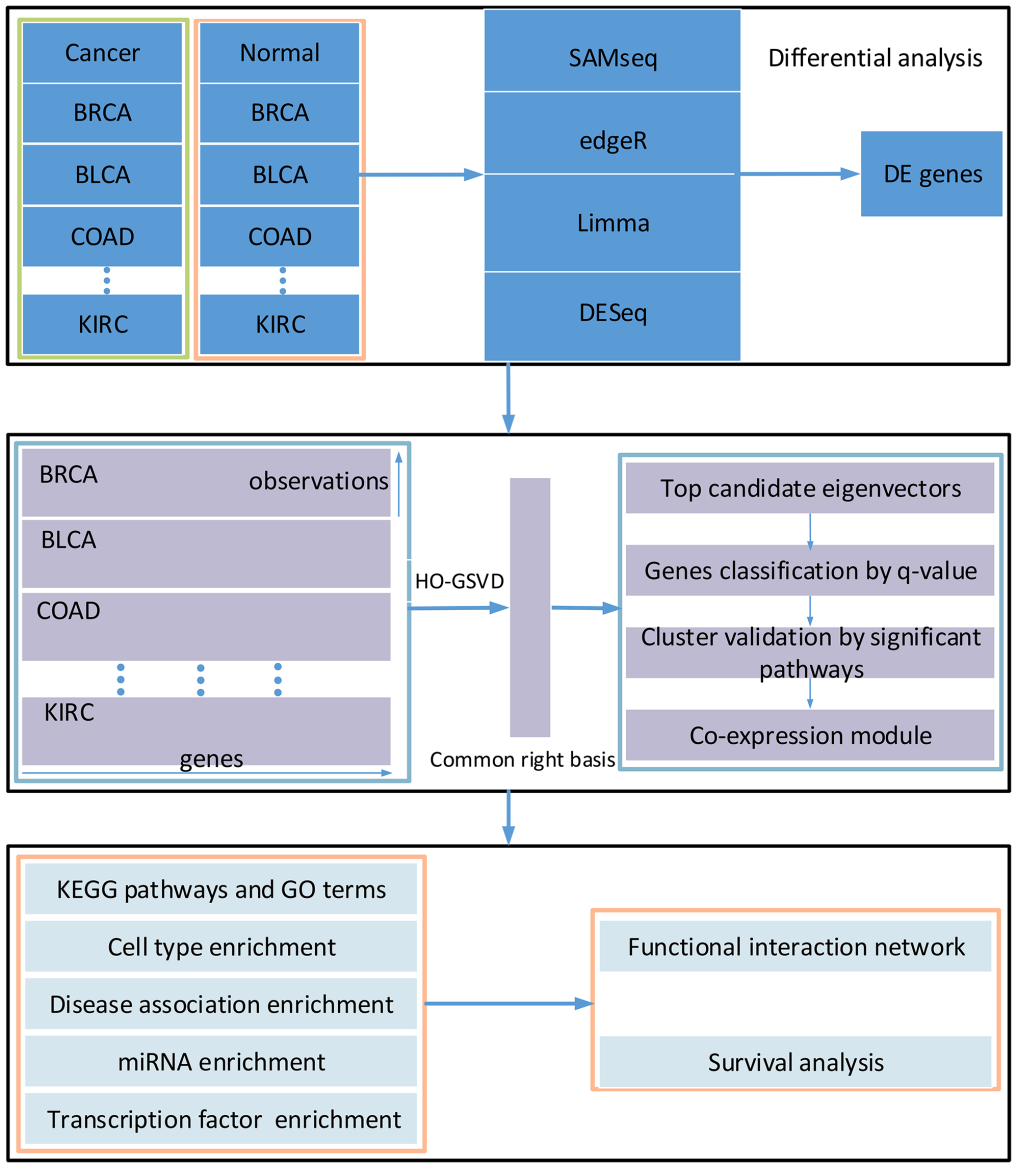
Overview of the workflow There are three main steps including gene differential expression analysis, identification of co-expression modules and significant enrichment analysis.

In this study, we applied the above four methods for differential expression analysis to get common genes in eight types of cancers through a three-step procedure. First, the differentially expressed gene set shared by four caner types was selected using one method. Second, the commonly expressed gene set selected by four methods was set as a candidate gene set. We then converted candidate gene IDs to the corresponding DAVID gene IDs and removed the non-mapped genes. The gene expression matrices of multiple cancers were decomposed by the HO-GSVD method for identifying the common modules across different cancers. We chose the vectors with top eigenvalues in the right basis matrix as candidates of co-expression genes. The co-expression genes were selected based on the assumption that a small proportion of genes in candidate vectors is highly similar. We used the DAVID tools to validate the functional significance of the modules. The gene modules involved in the significant pathways were retained for further analysis. Multiple types of enrichment analysis were performed, including gene ontology terms, KEGG pathways, cell type enrichment, disease association and miRNA and transcription factor enrichment analysis [15–17]. The functional interaction networks were constructed for the identified modules. The survival analysis was then applied for the prognostic properties of modules across cancers. By following the procedure shows in the Figure 1, we found that the co-expression gene modules are enriched with oncogenes and tumor suppressors, which play an important regulatory role in multiple cancers. The genes in the main- and sub-modules are closely associated with the prognosis of multiple cancers.

## RESULTS

### Identifying co-expression gene modules

**Figure 2:**
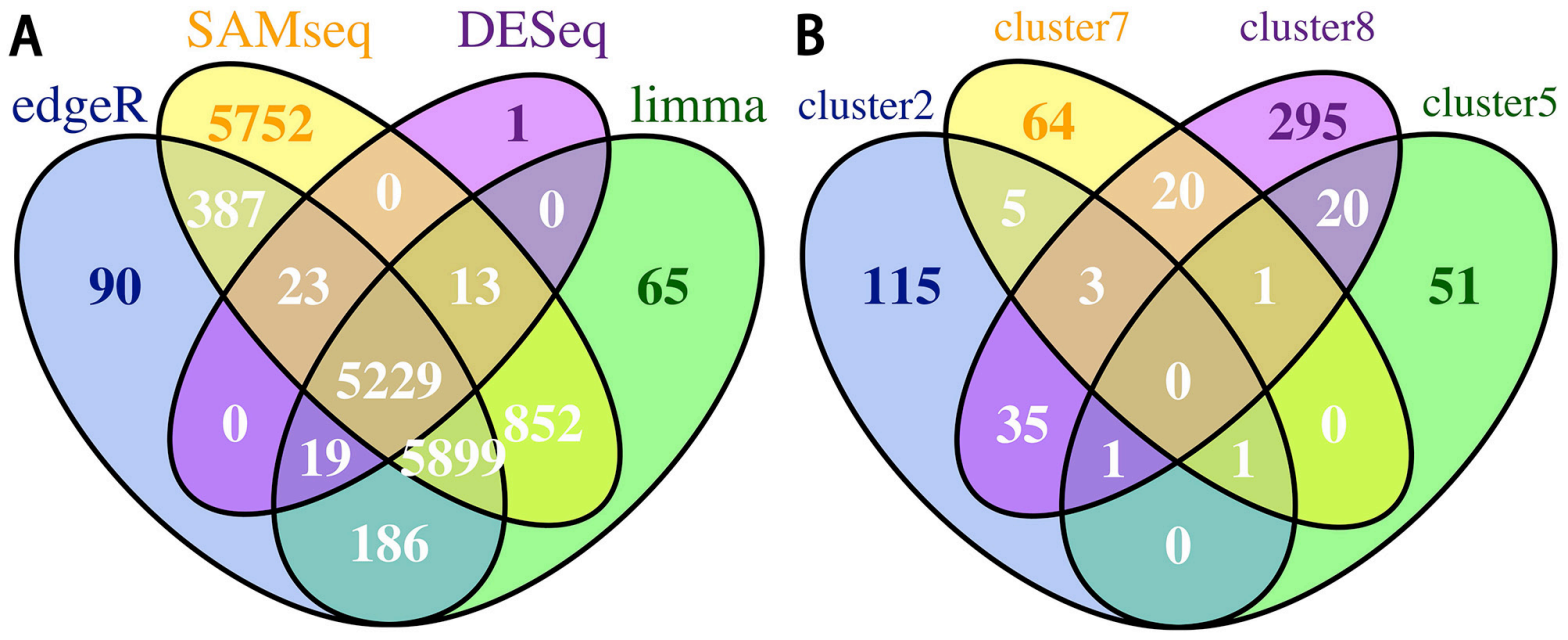
Identification of differentially expressed genes and co-expression modules **(A)** Venn diagram showing the overlap between differentially expressed genes selected by the four methods. **(B)** Venn diagram showing the overlap between the genes in the four co-expression modules.

We analyzed differentially expressed genes using the raw count data and constructed co-expression networks using the FPKM count data. 5229 differentially expressed genes were detected in breast invasive carcinoma (BRCA), kidney renal clear cell carcinoma (KIRC), prostate adenocarcinoma (PRAD), thyroid carcinoma (THCA), lung adenocarcinoma (LUAD), bladder urothelial carcinoma (BLCA), colon adenocarcinoma (COAD) and stomach adenocarcinoma (STAD) using limma, edgeR, DESeq, and SAMseq methods (Figure 2A). 4973 genes were used for construction of co-expression networks and pathway enrichment analysis. Five clusters with significant pathways were identified (Supplementary Table 1). The significant pathways enriched in the smallest cluster 10 were almost identical to the pathways in the cluster 5. Then five clusters except cluster 10 were kept for further analysis. The highly specific genes were included in each cluster. But there were a few genes shared by these clusters (Figure 2B). The results showed that these co-expression modules with specific genes may share similar biological information. To comprehensively investigate the characterization of the identified co-expression modules, we applied multiple types of enrichment analysis.

**Figure 3:**
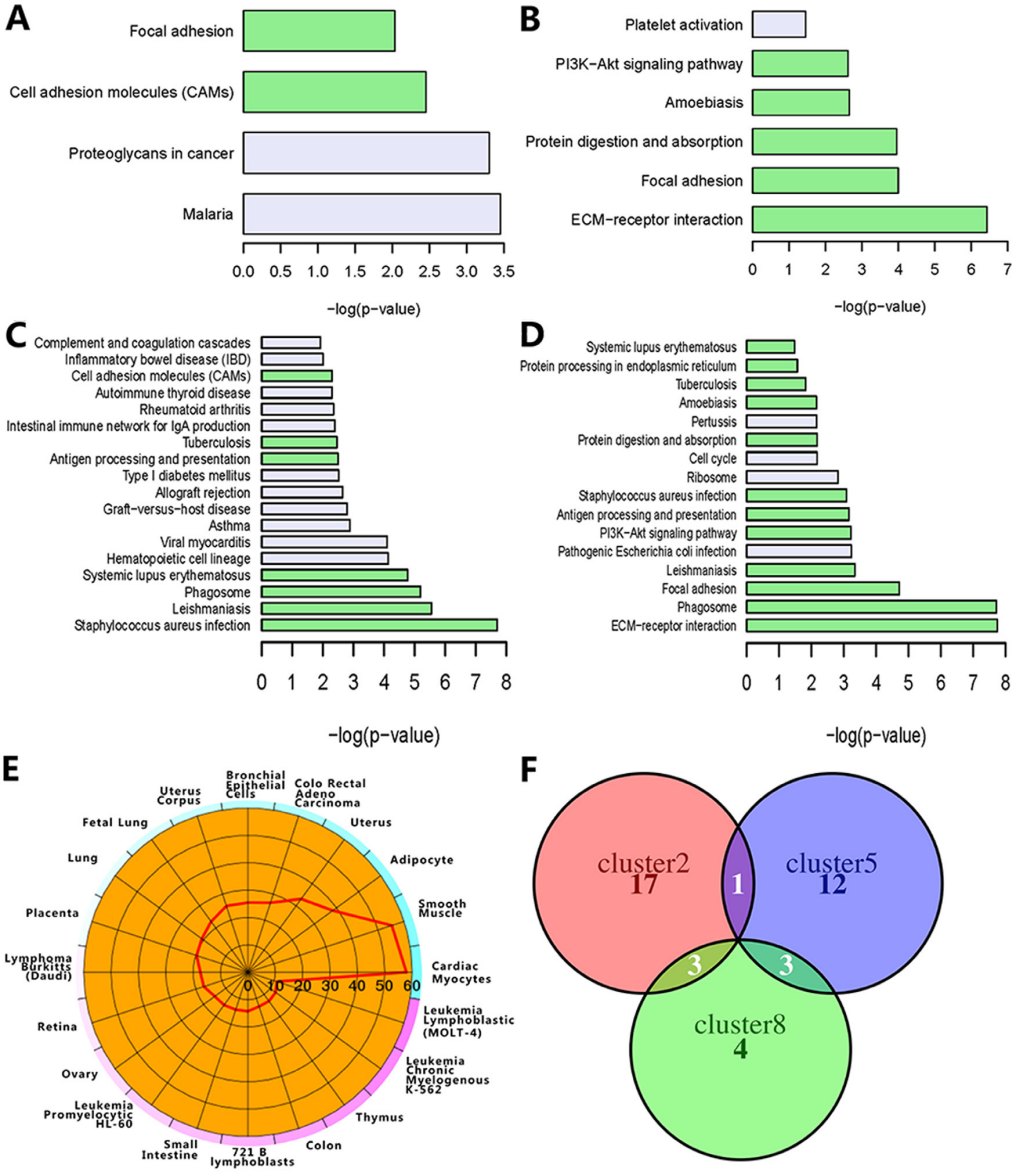
Enrichment of co-expression modules The four bar charts display the pathway enrichment results of cluster 2 **(A)**, cluster 5 **(B)**, cluster 7 **(C)** and cluster 8 **(D)**. The pathway shared by at least two cluster are colored with light green. The cell-type enrichment analysis of cluster 8 **(E)** and other clusters (Supplementary Figure 2) is shown as –log10 (Benjamini-Hochberg corrected p-value). The overlap of the members in the top 5-ranked miRNA families is shown as the Venn diagram **(F)**.

We applied functional enrichment analysis to identify the KEGG pathways (Figure 3A–3D) and Gene Ontology terms (Supplementary Table 2) in the four clusters. For cluster 5, there are only six significant KEGG pathways as shown in the Figure 3B. ECM-receptor interaction, focal adhesion and PI3K-Akt signaling pathways were presented in the cluster 8 and cluster 5, consistent with the previous results obtained by pan-cancer analysis [18]. Moreover, these pathways are related to the deregulation of cellular energetics [19]. Abnormal ECM affects cancer progression by directly promoting cellular transformation and metastasis [20]. Focal adhesion kinase, a protein tyrosine kinase, regulates cellular adhesion, motility, proliferation and survival in cancer cells, thereby promoting cancer progression and metastasis [21]. PI3K-Akt signaling pathway has been reported as one of the most important pathways in cancer metabolism and growth [22]. Figure 3C and Figure 3D show the enriched pathways in the cluster 7 and 8, respectively. Only focal adhesion was shared by the cluster 2, 5 and 8 (Figure 3A, 3B, 3D). These results indicated that the enriched pathways in the clusters are associated with tumorigenesis. To further evaluate whether the functional features in these clusters are also important in other cancers, we did analysis for another four cancers, including uterine corpus endometrial carcinoma (UCEC), head and neck squamous cell carcinoma (HNSC), rectum adenocarcinoma (READ) and liver hepatocellular carcinoma (LIHC) (Supplementary Figure 1). 8619 genes were identified. We converted the candidate gene IDs to the corresponding DAVID gene IDs. If two IDs were corresponding to the same gene, we removed the redundant one. Therefore, 490 redundant genes were removed and 8129 genes used for further analysis. We got two clusters with significant pathways (Supplementary Table 3) using HO-GSVD approach. We found that cluster 6 in the four cancers was highly enriched in focal adhesion $\left(p_{6}=5.7\text{e}-11\right)$, ECM-receptor interaction $\;(p_{6}=2.6\text{e}-10)$ and PI3K-Akt signaling $\left(p_{6}=2.7\text{e}-5\right)$ pathways. For the eight cancers, these clusters partially shared some KEGG pathways. We observed some similar patterns in the following results of the GO biological process (BP) enrichment analysis. The top-ranked five enriched BPs in the cluster 5 included collagen fibril organization $\left(p_{5}=3.5\text{e}-6\right)$, extracellular matrix organization $\left(p_{5}=4.5\text{e}-6\right)$, collagen catabolic process $\left(p_{5}=4.9\text{e}-5\right)$, skeletal system development $\left(p_{5}=3.2\text{e}-3\right)$ and cell adhesion $\left(p_{5}=1.3\text{e}-2\right)$. The top three enriched BPs in the cluster 8 were the same as those in the cluster 5. It was reported that the collagen fibrils reorganization within an extracellular matrix facilitated tumorigenesis and invasion [23]. We found that the BPs in the cluster 7 are mainly involved in T cell and immune response [24]. The highly enriched BPs in the cluster 2 are related to angiogenesis. In addition to cluster 7, these commonly enriched BPs are associated with extracellular matrix organization and cell adhesion. Similarity to the KEGG pathways, the corresponding enriched BPs in these clusters also play an important role in cancers. As expected, all the above results indicated that the identified clusters with biological functions related to cellular energetics, angiogenesis and anti-cancer immunity are important to the cancer development.

To further explore the cell specificity and disease association of the differentially expressed genes, we analyzed cell type enrichment (Cten) and disease association for the genes in four clusters (Supplementary Tables 4, 5). Enriched cell types in these clusters were closely associated with all eight cancer types, including BRCA, KIRC, PRAD, THCA, LUAD, BLCA, COAD, STAD. The top three cell types for the cluster 8 included cardiac myocytes (score as –log10 (Benjamini-Hochberg corrected p-value), score = 57), smooth muscle (score = 55) and adipocyte (score = 38) (Figure 3E). Adipocytes can support tumorigenesis by secreting adipokines and producing energy [25]. The immune cells in the cluster 7 and cluster 8 included CD14+ Monocytes, CD33+ Myeloid and BDCA4+ Dentritic Cells. CD14+ Monocytes (score = 4.88 in the cluster 7 and score = 7.81 in cluster 8) has been reported to affect dendritic cell differentiation and T-cell function in cancer patients [26]. These results indicated that the enriched cell types in the clusters were associated with tumor progression. Moreover, disease association analysis suggested that the genes in the four clusters were mainly enriched in cancer-related diseases with high significance (Supplementary Table 5, p≤8.39e−8). All the above results further suggested that the four clusters which play important roles in biological processes related to tumor progression and immunity are associated with multiple cancers.

To further demonstrate the potential regulatory mechanisms, we also analyzed miRNAs and transcription factors using the WebGestalt tool (Supplementary Tables 6, 7). Three of the top ranked miRNA families were identical in the cluster 2, 5 and 8, including mir-199, mir-29 and mir-200 (Figure 3F). Mir-199a was only presented in the cluster 2 and cluster 5 $\left(p_{2}=0.004,\;p_{5}=0.0035\right)$. Mir-29a, mir-29b and mir-29c $\left(p_{5}=0.0009,\;p_{8}=5.88\text{e}-5\right)$ in the cluster 5 and 8 are the mature members of the mir-29 family. Three of the main mir-200 family members $\left(p_{2}=0.0004,\;p_{8}=5.88\text{e}-5\right)$ in the cluster 2 and 8 have been reported to associate with tumor progression [27, 28]. The let-7 family of miRNAs $\left(p_{5}=0.0009\right)$ in the cluster 5 is involved in tumorigenesis [29]. We also found that some clusters were high significantly enriched with transcription factors. *FOXO4* gene was presented in the cluster 2, 5 and 7 $\left(p_{2}=2.82\text{e}-9,\;p_{5}=4.29\text{e}-5,\;p_{7}=0.0027\right)$ [30]. The clusters 2, 5 and 8 shared three transcription factors, including *E2A* $\left(p_{2}=3.66\text{e}-8,\;p_{5}=1.06\text{e}-9,\;p_{8}=7.38\text{e}-15\right)$, *NFAT* $\left(p_{2}=8.79\text{e}-11,\;p_{5}=2.38\text{e}-5,\;p_{8}=3.51\text{e}-17\right)$, and *MAZ* $\left(p_{2}=2.47\text{e}-8,\;p_{5}=2.40\text{e}-6,\;p_{8}=2.01\text{e}-15\right)$. *E2A*, *FOXO4*, *NFAT* and *MAZ* play important roles in tumor cell growth and metastasis in multiple cancers, such as colorectal cancer, breast cancer, prostate cancer and lung cancer [31–33]. Taken together, these results revealed that common regulators shared by the four clusters may cooperate with each other to regulate the biological processes of multiple cancers.

### Functional network analysis of individual co-expression modules

**Figure 4:**
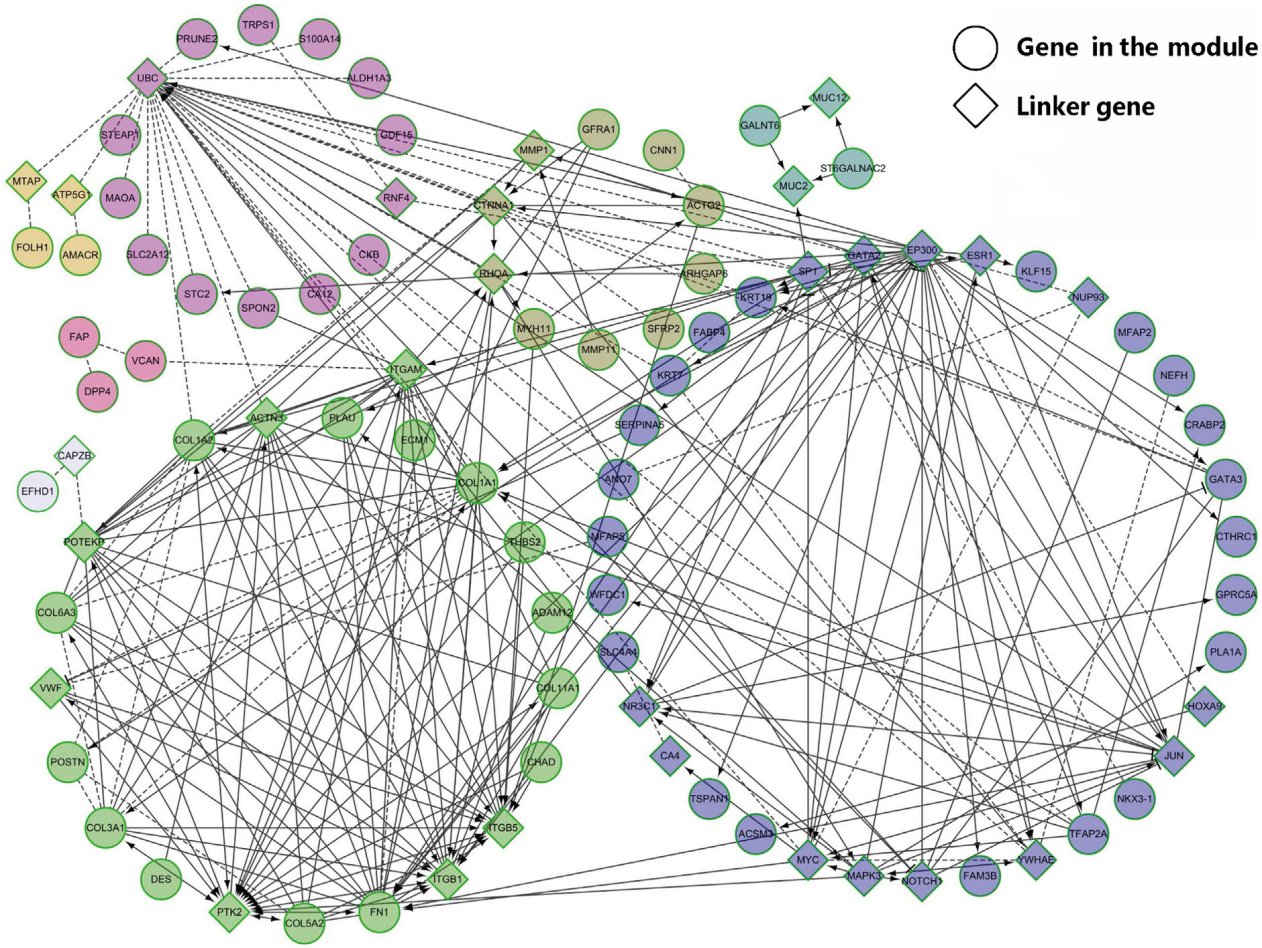
Network visualization of the cluster 5 The functional interaction network consists of eight sub-modules marked with different colors. The genes and link genes in the modules are represented as circles and diamonds, respectively.

To further analyze the characterization of single identified modules, we constructed the functional interaction networks. These networks were built based on human PPIs, fly PPIs, worm PPIs, yeast PPIs, domain interaction, Lee's Gene Expression, Prieto's Gene Expression, GO BP sharing and PPIs from GeneWays [34]. We found that the corresponding proportions of the genes linked to the functional interaction (FI) networks in the four modules were 80.63%, 83.78%, 76.60% and 87.20%, respectively (Supplementary Table 1). This result indicated that the four clusters were highly conserved at the functional protein level. According to the results from the above subsections, all clusters were closely associated with the eight cancers. Among of them, most of the enriched pathways in the cluster 5 were consistent with the previous pan-cancer outcomes [18], therefore, we did further functional analysis of the cluster 5-based FI network as shown in the Figure 4.

The FI network was clustered into small modules and eight modules were annotated as M1~M8 (Supplementary Table 8). We performed the enrichment analysis for GO BP and found that some high significantly BPs (FDR < 0.001) were consistent with the above results. Extracellular matrix disassembly, collagen catabolic process, extracellular matrix organization, collagen fibril organization, skeletal system development and cell adhesion were enriched in the M2. The BPs in the M7 are associated with the negative regulation of extracellular matrix disassembly and endothelial cell migration. Endothelial cell migration has been reported to contribute the entry of cancer cells into the circulatory system [35]. There were two BPs in the M5, including O-glycan processing and protein O-linked glycosylation. It has been reported that alterations in glycosylation impacted cell cycle and may support neoplastic progression [36]. Interestingly, M1 had only one enriched pathway, that is, the notch signaling pathway. Notch signaling pathway plays an important in regulating stem cell self-renewal and the pathogenesis of breast cancer [37]. The most enriched pathway in the M2 was ECM-receptor interaction. Phenylalanine metabolism was the top enriched pathway in the M3. Nicotinic acetylcholine receptor signaling pathway in M4 and amino acid metabolism pathway in M6 are associated with cancer growth [38–40]. These results indicated that distinct BPs enriched in different sub-modules contribute to the development of cancers.

We then defined an individual gene in the modules with at least ten neighbors as one hub gene, and obtained 20 hub genes, including 15 linker genes and 5 module genes. Over half of the hub genes were enriched in the M2, including *FN1*, *COL1A1*, *COL1A2*, *COL3A1* and *COL6A3*. *FN1* is a FDA-approved drug target gene against cancer [41, 42]. *COL1A1*, *COL1A2*, *COL3A1* and *COL6A3* are members of the collagen family. These five genes were enriched in pathways such as ECM-receptor interaction, protein digestion and absorption, integrin signaling pathway and PI3K-Akt signaling pathway. *DES*, *THBS2*, *PLAU* and *POSTN* in the M2 have been associated with multiple cancers [43–50]. *DES* encodes a muscle-specific class III intermediate filament and is related with colorectal cancer, breast cancer, prostate cancer, kidney cancer and lung cancer [43–46]. As the member of *THBS* family, *THBS2* plays an important role in cancer progression [47]. *PLAU* is involved in cancer cell migration [48]. The protein encoded by *POSTN* has been reported to function in cancer stem cell [49, 50].

### Survival analysis of gene co-expression modules

**Figure 5:**
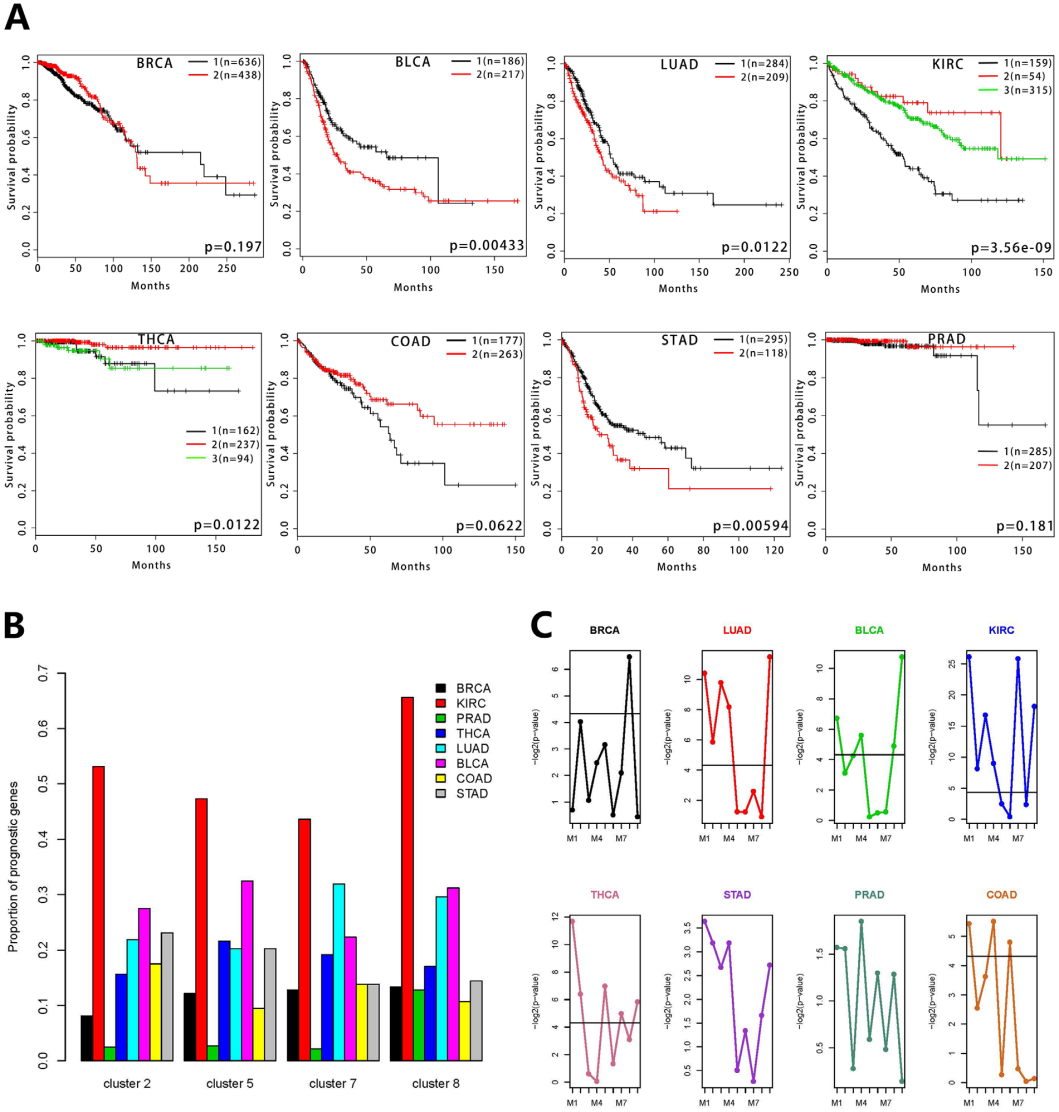
Survival analysis of gene co-expression modules for 8 types of cancers **(A)** Survival analysis of the cluster 5 using Kaplan-Meier curves. The calculation of the log-rank p-values is based on the split of patient groups using non-negative matrix factorization. The number of patients in each group is also labeled in each panel. **(B)** The distributions of prognostic genes for the eight cancer types in each cluster. The y-axis represents the gene proportion of each cancer in the corresponding cluster. The eight cancers are marked with different colors. **(C)** The differential significance distributions of nine sub-modules for each cancer. The y-axis represents the log-rank p-value and the x-axis is annotated with the sub-modules grouped by the network clustering. The solid line represents –log_2_(0.05).

To further test the clinical significance of the co-expressed gene clusters, we did survival analysis for the eight types of cancers. The patients were clustered into different groups using non-negative matrix factorization (NMF). The cluster 2 and cluster 5 were able to predict patient survival of various cancers, including KIRC, THCA, LUAD, BLCA, COAD and STAD (Figure 5A, Supplementary Figure 3). The cluster 7 and cluster 8 could predict survival of LUAD, KIRC and BLCA cancer patients (Supplementary Figures 4, 5). The cox models were used to identify prognostic genes in various cancers. The distinct proportions of prognostic genes in the eight cancers were observed in the four clusters (Figure 5B). Among all of the clusters, KIRC contains the largest proportion of prognostic genes. We also carried out the survival analysis on the eight sub-modules in the cluster 5, as well as the module 9 containing the genes that weren't connected to the networks in the cluster 5 (Supplementary Tables 8, 9). The patients were grouped using the k-means method when the number of genes in the sub-module was one. In the survival analysis of individual sub-modules, all sub-modules showed statistically significant differences in survival probabilities (Figure 5C). M1, M2, M4 and M9 were significantly associated with the patients’ survival in at least three cancers. But only one sub-module showed a significant difference in the survival analysis of BRCA and none of the sub-modules was significant in PRAD. Analysis of STAD showed that individual sub-modules couldn't predict these patients’ survival but the corresponding cluster 5 had the predictive power. The results of the sub-modules in the survival analysis were mostly consistent with the cluster 5. All these results revealed that the sub-modules associated with known biological processes cooperate with each other to contribute to the prognosis in multiple cancers.

**Figure 6:**
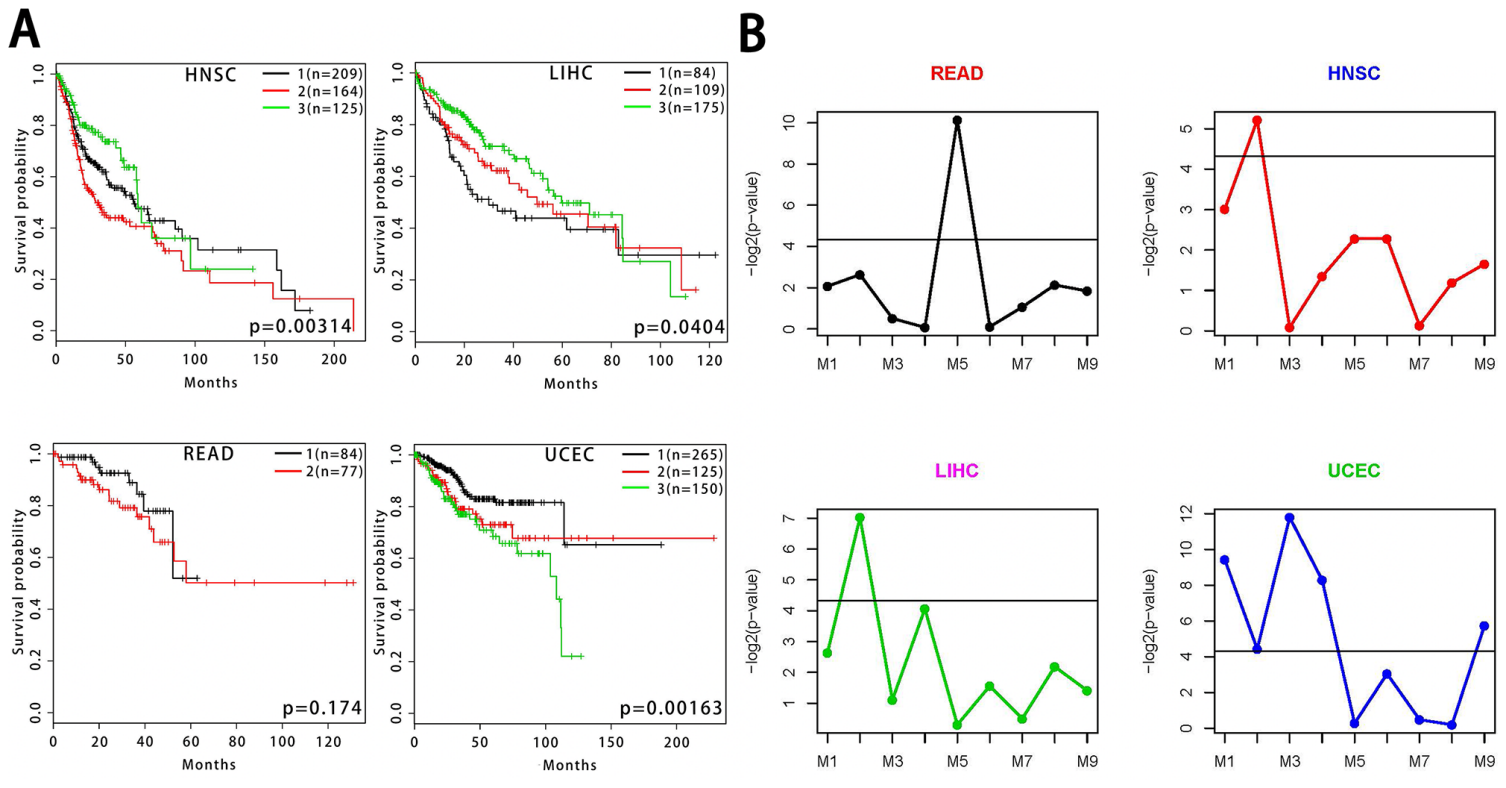
Survival analysis of gene co-expression modules for HNSC, LIHC, READ and UCEC **(A)** Survival analysis of the cluster 5 using Kaplan-Meier curves for HNSC, LIHC, READ and UCEC. **(B)** The differential significance distributions of nine sub-modules for each cancer.

In order to further validate the prognosis of cluster 5 in multiple cancers, we performed survival analysis on HNSC, READ, LIHC and UCEC. The results showed that the cluster 5 was able to predict patient survival in HNSC, LIHC and UCEC (Figure 6A), but not in READ. The corresponding sub-modules obtained from the functional network analysis had statistically significant differences in survival analysis of the four cancers (Figure 6B). M2 had predictive power for patient survival in three cancers. We also applied survival analysis on other eight cancers, including glioblastoma multiforme (GBM), brain lower grade glioma (LGG), ovarian serous cystadenocarcinoma (OV), skin cutaneous melanoma (SKCM), adrenocortical carcinoma (ACC), cervical squamous cell carcinoma and endocervical adenocarcinoma (CESC), kidney renal papillary cell carcinoma (KIRP) and lung squamous cell carcinoma (LUSC). As shown in the Supplementary Figure 6, the cluster 5 can predict patients’ survival of LGG, SKCM, ACC and KIRP cancers. These results confirmed the prognostic ability of cluster 5 in 7 cancer types. We also applied survival analysis on the above twelve cancer types for the cluster 2, 7 and 8. All of the three clusters can predict patients’ survival of LIHC, UCEC, LGG, SKCM, ACC and KIRP cancers. Additionally, cluster 2 can predict the survival of HNSC and CESC cancer patients.

## DISCUSSION

In this study, we applied four methods to analyze the data and obtained 4973 differentially expressed genes shared by the eight cancers. We constructed the co-expression network using HO-GSVD and identified modules and associated pathways. Some of these pathways have been reported in the pan-cancer analysis [18], such as focal adhesion, PI3K-Akt signaling pathway, ECM-receptor interaction and Systemic lupus erythematosus. We constructed the functional interaction networks for further analyzing individual co-expression modules. These sub-modules were enriched with different pathways and cooperated with each other across cancers. We found that these modules are associated with patients’ survival in multiple cancers. In addition, the individual sub-modules in various cancers had different prognostic capability.

Gene differential expression analysis is a commonly used method for identifying differentially expressed genes without considering the relationship between genes. There is no consensus on the best method for differential expression analysis [14, 51]. Limma, edgeR, DESeq, and SAMseq have been commonly used for gene differential expression analysis. Each of them has advantages and disadvantages. The limma method, based on linear models and the voom transformation, is developed for analyzing RNA-seq data [52]. The negative binomial model and empirical Bayes methods in edgeR are used to detect differential gene expression, and then the gene-wise dispersions is estimated by conditional maximum likelihood [10]. DESeq models the count data using the negative binomial distribution and estimates the mean-variance relationship of each gene. In contrast to edgeR, it allows a widely data-driven parameter in the statistical test [13]. SAMseq, a non-parametric method, uses the Wilcoxon rank statistic and resampling procedure to identify differential expressed genes [12]. edgeR becomes liberal for small sample sizes with default settings to a certain extent and keeps a better balance between speed and accuracy than DESeq [14, 51]. Limma and DESeq are described as the safest choices in some cases in terms of the consistency of differentially expressed genes when analyzing the complete data. SAMseq can identify a large number of genes that are usually not detected with the other methods [53]. The combination of these methods can compensate for the shortcomings of a single method. The results of differential expression analysis obtained using these four methods are robust. We chose the co-expression networks to analysis cancer-related genes on the system level. Then we applied the simple framework of HO-GSVD to identify co-expression modules, which has been proved to be a simple, parameter-free and reproducible method. The results in the functional enrichment analysis showed the all the identified modules may play regulatory roles in multiple cancers. We found that the enriched BPs and pathways in the three clusters are associated with the cancer hallmarks including deregulating cellular energetics and inducing angiogenesis [54]. The enriched pathways in the cluster 5 and 8 are related to deregulating cellular energetics, including focal adhesion, PI3K-Akt signaling pathway and ECM-receptor interaction. Focal Adhesion Kinase through ECM promotes the activation of PI3K-AKT signaling [55]. Then P13K-AKT signaling pathway increases glycolysis in metabolic processes [19]. The enriched BPs in the cluster 2 are related to inducing angiogenesis, such as platelet degranulation, positive regulation of angiogenesis, leukocyte migration and angiogenesis. Angiogenesis plays an important role during macroscopic and microscopic neoplastic progression [54]. The microenvironment-related BPs in the cluster 7 are closely associated with anti-cancer immunity. Not surprisingly, we found that the genes in the modules were implicated in diseases, such as neoplastic processes, immune system diseases, cancer or viral infections and neovascularization. Some miRNA and transcription factors were also enriched in the modules. The mir-200 family has been identified as a biomarker in cancer [56]. Mir-199a has been associated with various cancers, including kidney, breast, bladder, bronchial squamous and stomach cancers [57–61]. Studies indicate that mir-29 family members may cooperatively or separately contribute to the development of breast and colon cancer [62]. *FOXO4* is reported to suppress tumor proliferation and metastasis in stomach carcinoma, and its clinical significance is observed in multiple cancers [63–65]. The *NFAT*-related roles have been studied in the tumor microenvironment [33]. *MAZ* promotes the tumor progression in glioblastoma, breast cancer, prostate cancer and hepatocellular cancer [31, 32]. The regulatory and clinical significance of *E2A* are studied in colorectal cancer [66]. Our study indicates that the genes in the identified modules play a cooperate role in multiple cancers through miRNA and transcription factors.

The sub-modules in the cluster 5 were analyzed for further understanding the regulatory mechanism in multiple cancers. We found that some genes in sub-modules play vital roles in multiple cancers, such as tumor suppressors and oncogenes. There was only one pathway in the largest sub-module M1 but the sub-module was highly enriched in cancer-related genes. Hub genes enriched in M2 have been identified to play a prognostic role in multiple cancers. Genes in M3 are involved in metastasis and prognosis. These sub-modules may help in discover new genes related with multiple cancers.

The identified modules showed different predictive power in prognosis of cancers. Some modules were able to predict survival in six cancers, such as cluster 2 and cluster 5. These modules were not significantly related to survival in BRCA and PRAD. Sample size and the number of deaths are two important factors in survival analysis. There exists the unbalanced number of genes involved in multiple cancers. These factors may result in the poor performance of prognostic capability. Some sub-modules in the cluster 5 showed the statistical significance of survival analysis in at least three cancers, such as M1, M2, M4 and M9. Other sub-modules also revealed the prognostic capability in different cancers. The prognosis of cluster 5 was validated in other cancers. Importantly, the cluster 5 could be prognostic in 12 cancer types in total.

In summary, we identified the functional modules and co-expression networks for the systematic analysis of the carcinogenic properties and regulatory mechanisms of multiple cancer. Further analysis indicates that these co-expression modules have a strong ability in predicting the survival of cancer patients. The results will be helpful in identifying new targets associated with cancer treatment. Our results will be valuable in cancer-related gene function annotation, and for the evaluation of cancer patients’ prognosis.

## MATERIALS AND METHODS

### Differential gene expression analysis

We downloaded TCGA gene expression data from the Gene Expression Omnibus under accession number GSE62944. Eight types of cancers were used to construct the co-expression network, including BRCA, KIRC, PRAD, THCA, LUAD, BLCA, COAD and STAD. After the construction of the co-expression network, we also analyzed UCEC, HNSC, READ and LIHC for further assessing the functional significance of the modules (Supplementary Table 9).

Four methods were used for differential gene expression analysis, including limma, edgeR, DESeq, and SAMseq. The sets of differentially expressed genes from each cancer were pooled together to increase the statistical power.

The genes with multiple test corrected p-value < 0.05 were considered to be significantly differentially expressed. For SAMseq, the number of permutations used to estimate false discovery rates was set to 200 and the number of resamples used to construct test statistic was set to 100. For limma, we used the TMM method of the edgeR package and the voom transformation.

We proposed a three-step procedure for selecting the common genes shared by the eight cancer types (BRCA, KIRC, PRAD, THCA, LUAD, BLCA, COAD, STAD). First, genes which were significantly differentially expressed in at least four types of cancers analyzed by one method were clustered into one gene set. Second, genes shared by all four gene sets obtained by four methods were selected as a candidate gene set. At last, the genes that were not mapped to the corresponding DAVID genes were removed. We detected genes shared by UCEC, HNSC, READ, and LIHC cancers by following the rules similar to the above procedure, but with modification. Three methods (edgeR, SAMseq, and Limma) were used and significant genes shared by at least two cancer types with one method were clustered in one gene set.

### Co-expression network construction

The gene expression data expressed as fragments per kilobase of exon per million reads mapped (FPKM) and normalized on log2 scale were represented by matrices. We then used HO-GSVD to extract common modules through matrix decomposition. The basic idea behind this approach is using spectral decomposition to identify common structures (subnetworks) in multiple datasets.

For the tth cancer (t=1, 2, …, T), the input data $D_{t}\in R^{n_{t}\times p}\;$ is the real matrix represented by the rows denoted by the samples $n_{t}$ and the columns denoted by the genes p. The mathematical form of the HO-GSVD framework of T real matrices is given by

$$D_{1}=U_{1}\Sigma_{1}V^{T},\;D_{2}=U_{2}\Sigma_{2}V^{T},\;\ldots ,D_{T}=U_{T}\Sigma_{T}V^{T}\;$$ (1)

where $U_{t}\in R^{n_{t}\times p}$ is composed of normalized left basis vectors, $\Sigma_{t}\in R^{p\times p}\;$ is a non-negative diagonal matrix consisted of the higher-order generalized singular values and $V\in R^{p\times p}$ is composed of normalized right basis vectors. As the previous method [8], the right basis vectors V were defined as the solution of the eigensystem of the matrix S:

$$S=\frac{1}{T(T-1)}\overset{T-1}{\underset{t=1}{\sum }}\overset{T}{\underset{r=t+1}{\sum }}(E_{t}E_{r}^{-1}+E_{r}E_{t}^{-1})\;$$ (2)

where the covariance matrix $E_{t}=D_{t}^{T}D_{t}$ can be treated as the co-expression matrix. Importantly, the common HO-GSVD subspace is spanned by the right basis vectors V. Then we used the right basis vectors to select common structures shared by all cancer types. The advantage of this approach for identification of co-expressed structures across cancer types is able to reproduce accurately common structures without relying on any predefined parameters.

Based on the eigenvalues of the eigen-decomposition of S, we chose the top ten eigenvectors to identify co-expression gene modules. A small part of the genes is supposed to have significantly similarity with each other and these genes can be regarded as the co-expression genes. Similarly to the strategy used in [67], the selected eigenvectors were decomposed into two components and modeled using Gaussian Mixture Model (GMM). In addition, the small weight component of bimodal distribution can identify the small proportional genes with high similarity. We calculated the tail area-based false discover rate (q-value) using the R package fdrtool to identify co-expression genes. Then we clustered the genes into the co-expression gene module through the cut-off of q-value (0.001).

### Enrichment analysis

Functional enrichment analysis of the co-expressions gene modules was performed using DAVID [15]. Benjamini-Hochberg method was used for the multiple test correction of p-values. KEGG pathways and Gene Ontology terms with Benjamini-Hochberg corrected p-value less than 0.05 were considered as significantly enriched. To validate the functional significance of modules, only gene modules involved in significant pathways were retained for further analysis. The cell type enrichment was analyzed using Cten [17]. Disease association analysis was conducted using the gene set analysis toolkit WebGestalt. In addition, the enrichment of miRNAs and transcription factors was performed using WebGestalt [16].

### Functional network analysis

We used module genes to construct the functional interaction network within the Cytoscape FI plugin [34]. Linker genes were used to maximize the number of connected genes in the module. Then we clustered the FI network into small modules. The functions of small modules were analyzed for GO terms and pathway enrichment.

### Survival analysis

We downloaded overall survival data from the Firehose. The patients with multiple repeated samples were not included in the survival analysis. The R package ‘survival’ was used for the Cox proportional hazards (PH) model and Kaplan-Meier survival analysis. To detect prognostic genes, we calculated P-values based on the raw Wald test for the Cox PH model. Based on the expression of module genes, we classified tumor samples into clusters through NMF [68]. We then estimated the significance of differences between different clusters with patients’ survival using log-rank test of the Kaplan-Meier method. We also performed survival analysis on small modules generated from the FI network.

## SUPPLEMENTARY MATERIALS FIGURES AND TABLES











## Abbreviations

DAVID: the Database for Annotation, Visualization, and Integrated Discovery
TCGA: The Cancer Genome Atlas
KEGG: Kyoto Encyclopedia of Genes and Genomes
