# Supplementary material for: Identification of cancer prognosis-associated functional modules using differential co-expression networks
Source: Oncotarget. 2017 Dec 4;8(68):112928–41. doi: 10.18632/oncotarget.22878 (PMC5762563; doi:10.18632/oncotarget.22878)
Supplement: Supplementary file 1 [file oncotarget-08-112928-s001.pdf]

Identification of cancer prognosis-associated functional modules using differential co-expression networks

SUPPLEMENTARY MATERIALS

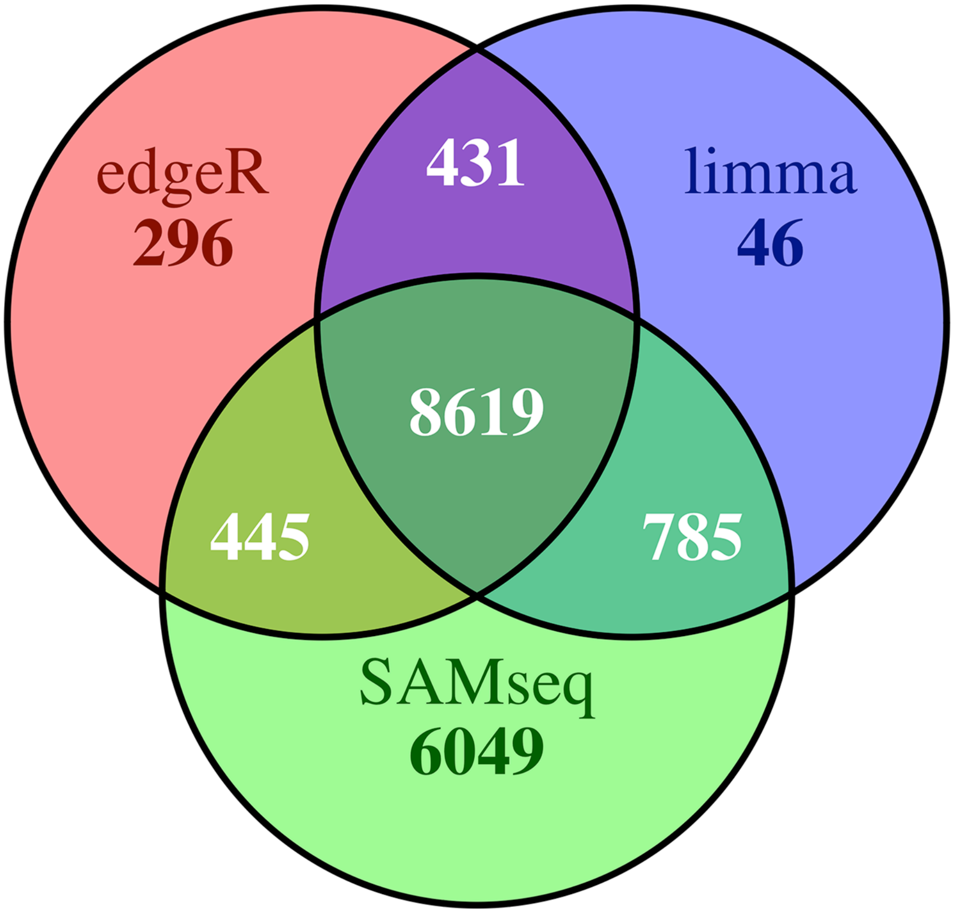

Supplementary Figure 1: Identification of differentially expressed genes for HNSC, LIHC, READ and UCEC. Venn diagram showing the overlap between differentially expressed genes selected by each method.

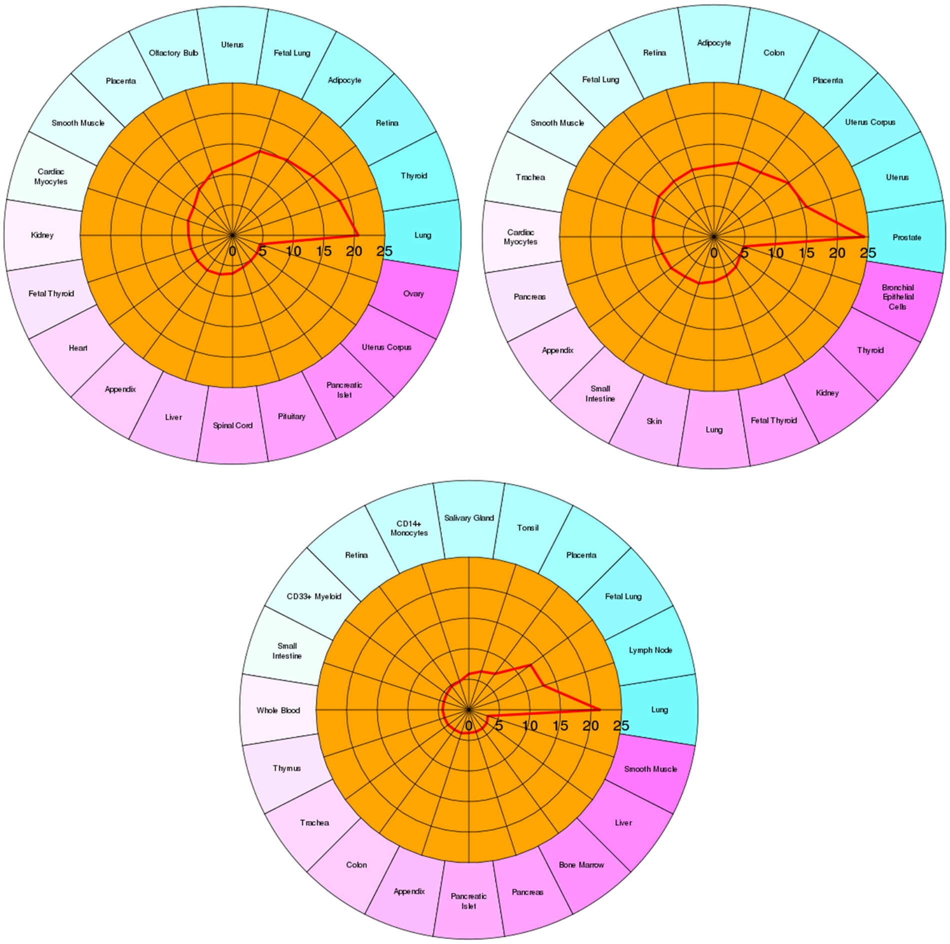

Supplementary Figure 2: Cell type enrichment of co-expression modules. The cell-type enrichment analysis of cluster 2 (upper left-hand corner), cluster 5 (upper right-hand corner), cluster 7 (bottom) is shown in as –log10 (Benjamini-Hochberg corrected p-value).

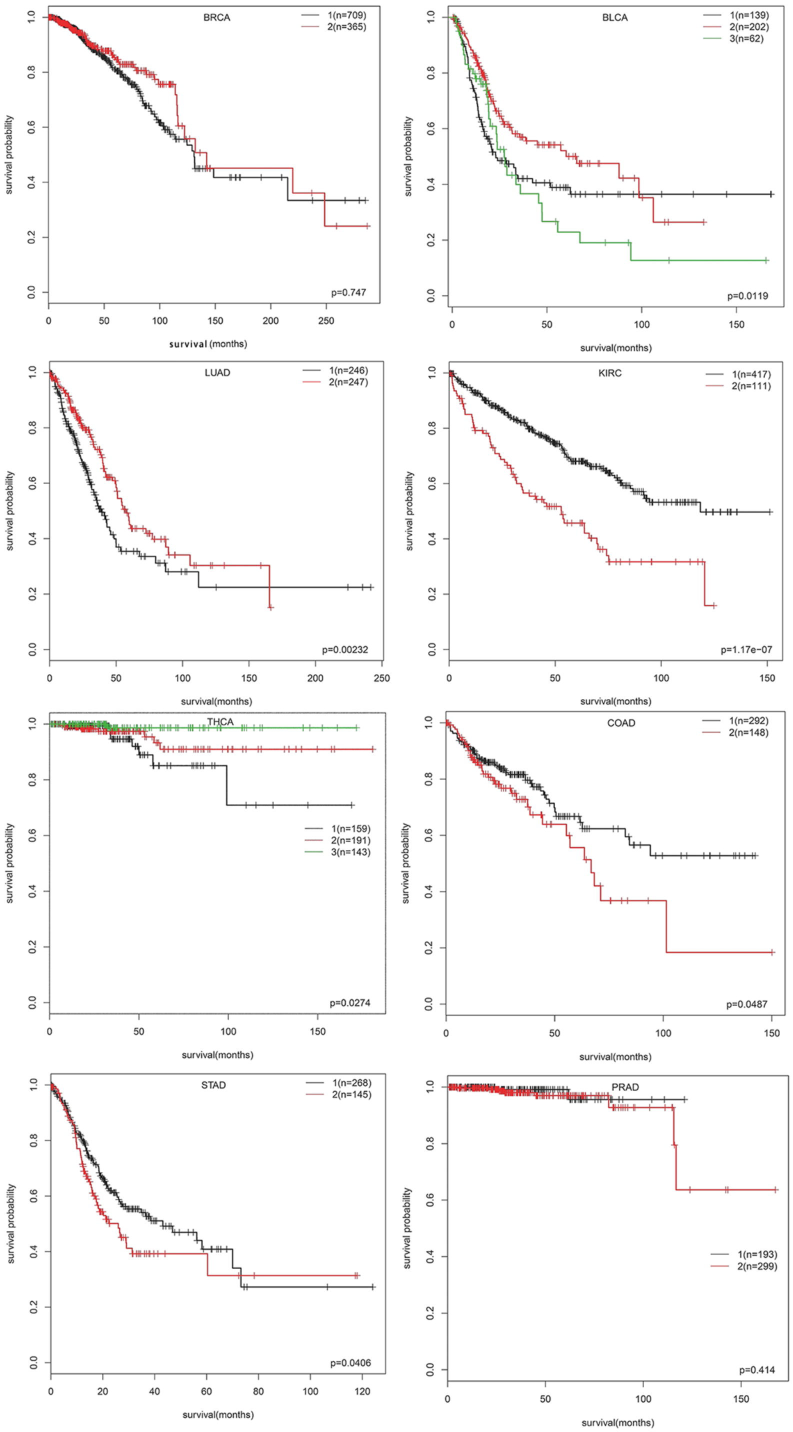

Supplementary Figure 3: Survival analysis of the cluster 2 for 8 types of cancers. Kaplan-Meier curves are used in survival analysis. The calculation of the log-rank p-values are based on the split of patient groups using non-negative matrix factorization. The number of patients in each group is also labeled in each panel.

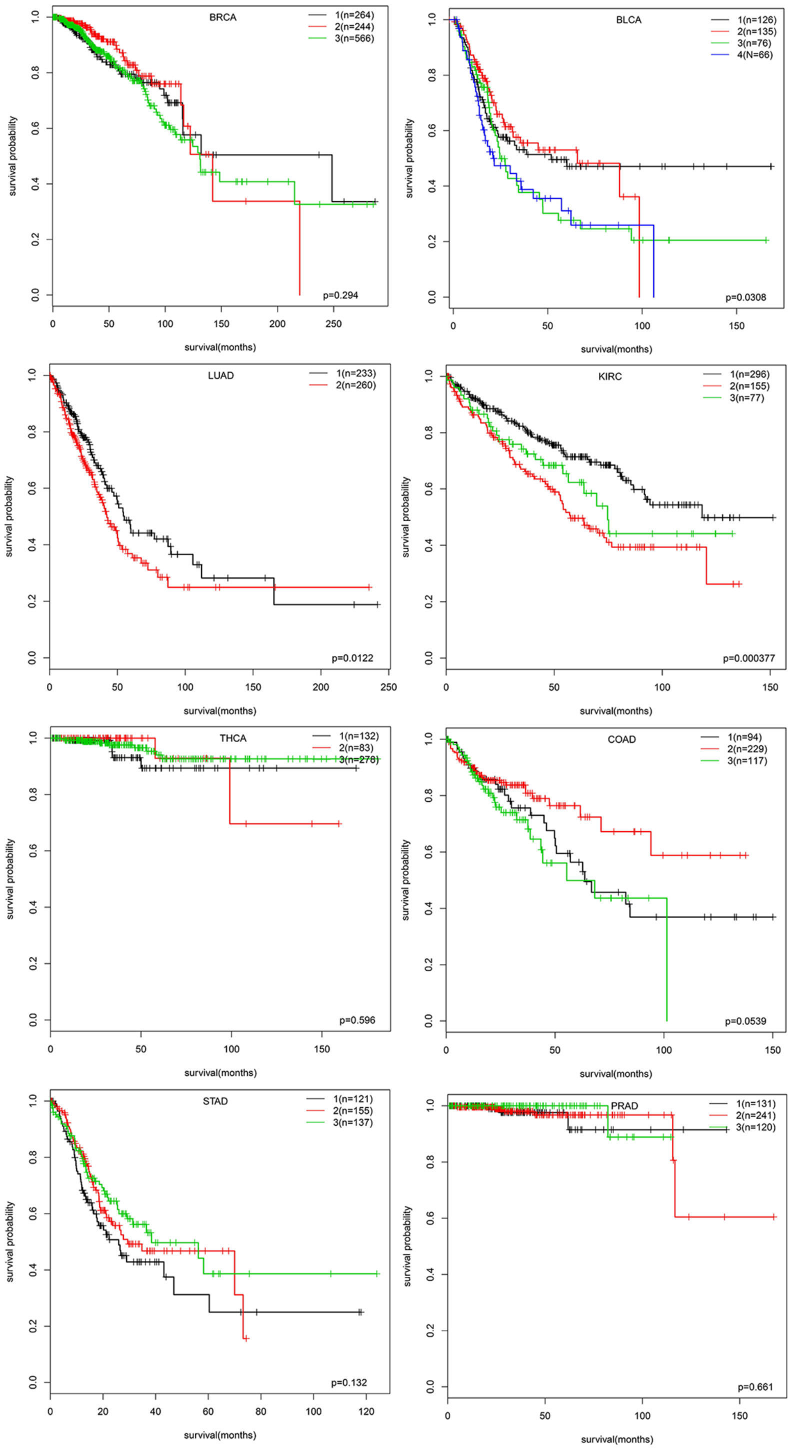

Supplementary Figure 4: Survival analysis of the cluster 7 for 8 types of cancers. Kaplan-Meier curves are used in survival analysis. The calculation of the log-rank p-values are based on the split of patient groups using non-negative matrix factorization. The number of patients in each group is also labeled in each panel.

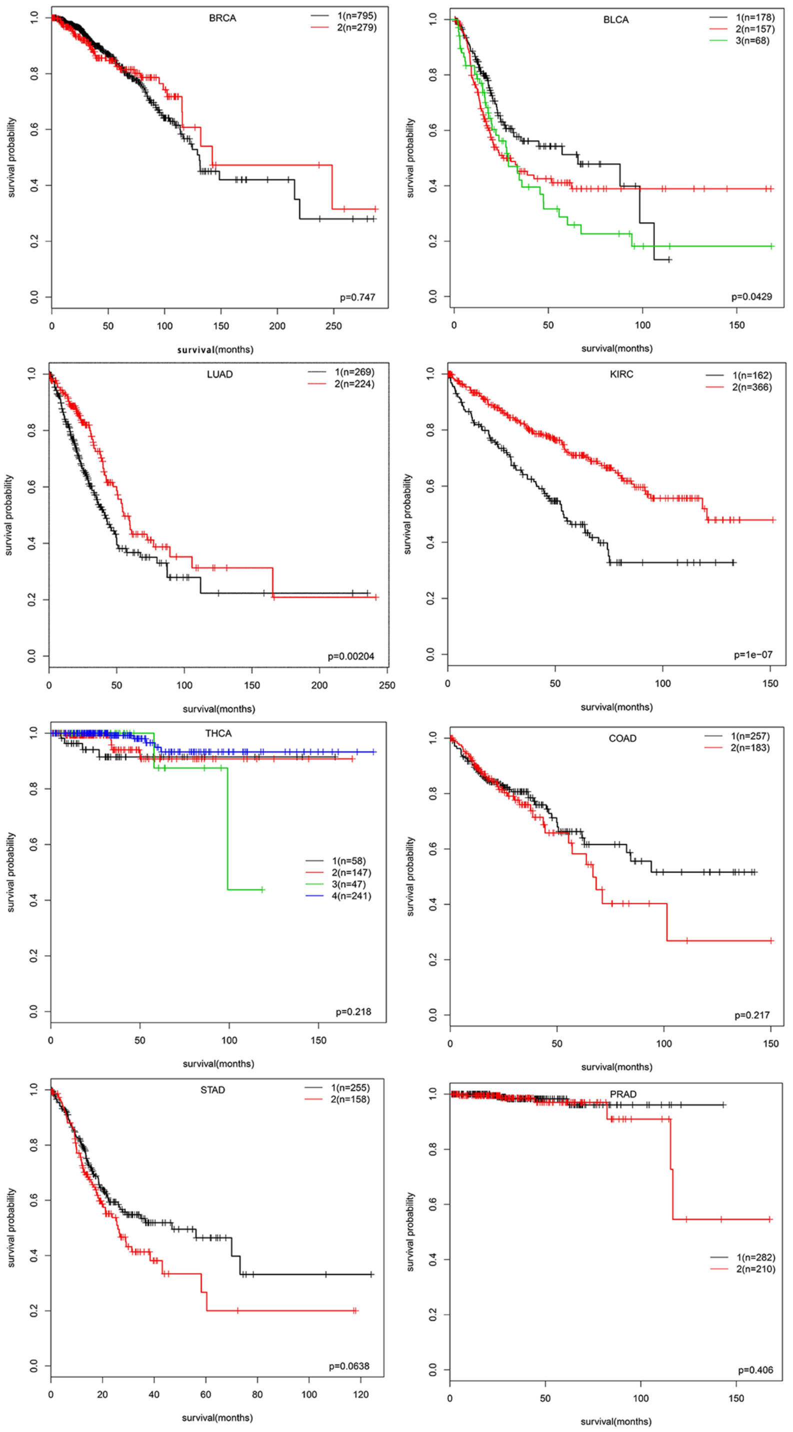

Supplementary Figure 5: Survival analysis of the cluster 8 for 8 types of cancers. Kaplan-Meier curves are used in survival analysis. The calculation of the log-rank p-values are based on the split of patient groups using non-negative matrix factorization. The number of patients in each group is also labeled in each panel.

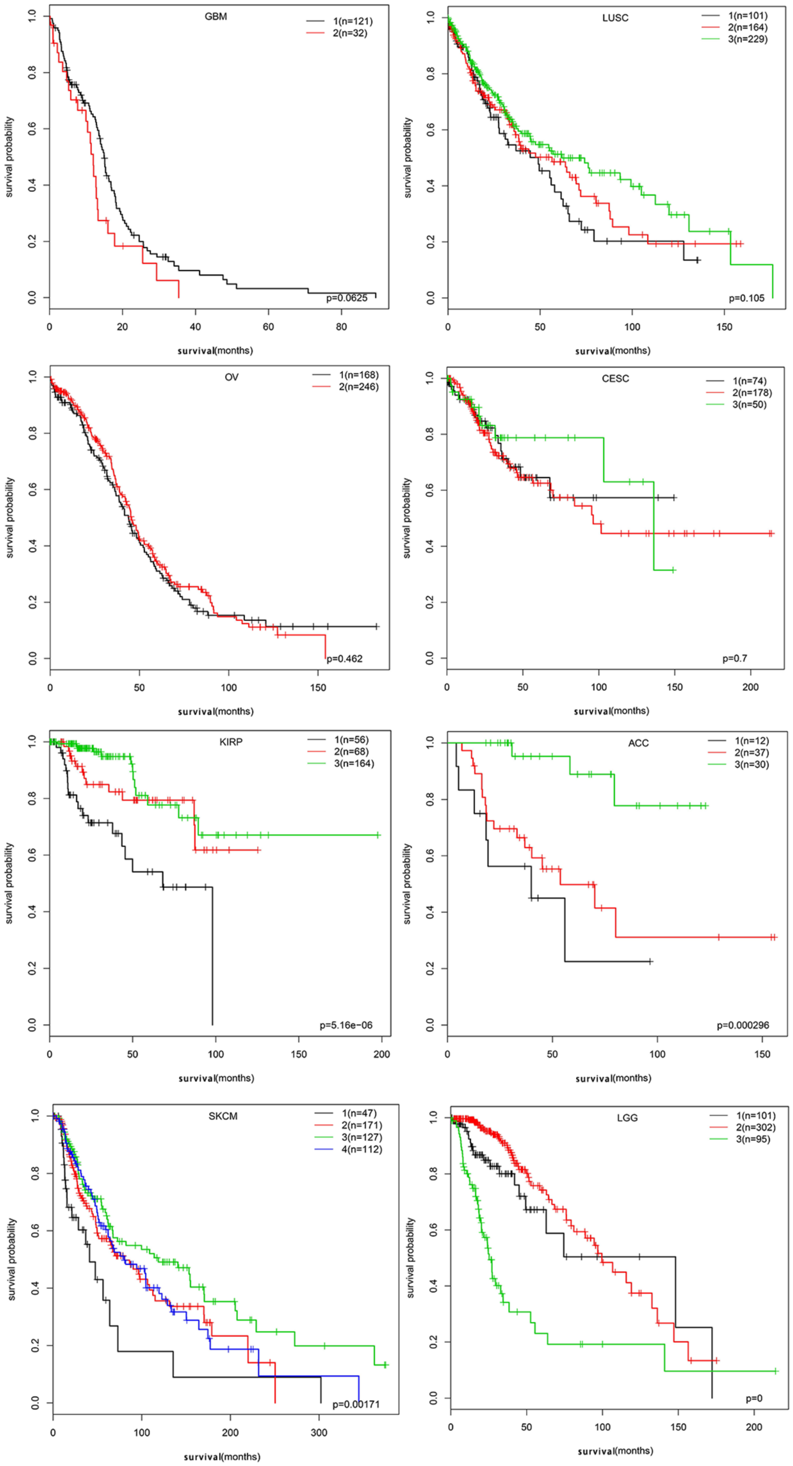

Supplementary Figure 6: Survival analysis of the cluster 5 for other 8 types of cancers. Kaplan-Meier curves are used in survival analysis for GBM, LUSC, OV, CESC, KIRP, ACC, SKCM and LGG. The calculation of the log-rank p-values are based on the split of patient groups using non-negative matrix factorization. The number of patients in each group is also labeled in each panel.

Supplementary Table 1: Number of genes in the cluster as well as number of genes used in the corresponding FI network

Module	Genes in the cluster	Genes in the FI network
Cluster 2	160	129
Cluster 5	74	62
Cluster 7	94	72
Cluster 8	375	327
Cluster 10	30	

Four clusters were identified in the eight cancer types.

Supplementary Table 2: GO BPs of the four clusters

See Supplementary File 1

Supplementary Table 3: Number of genes in the cluster

Module	Genes in the cluster
Cluster 4	279
Cluster 6	128

Two clusters were identified in the four cancer types.

Supplementary Table 4: Cell type enrichment of the four clusters. The score is–log10 (Benjamini-Hochberg corrected p-value)

See Supplementary File 2

Supplementary Table 5: The enriched diseases in the four clusters

	Disease	Benjamini-Hochberg corrected p-value
Cluster 2	Neovascularization, Pathologic	7.23E-23
	Neoplastic Processes	5.97E-22
	Carcinoma	5.97E-22
	Neoplasms	1.32E-19
	Metaplasia	3.94E-19
	Adhesion	1.93E-17
	epithelial cancers	4.32E-17
	Neoplasm Metastasis	4.32E-17
	Vascular Diseases	4.16E-16
	cancer or viral infections	1.36E-15
Cluster 5	cancer or viral infections	1.17E-10
	Neoplasms	1.55E-10
	Carcinoma	5.07E-10
	epithelial cancers	5.26E-10
	Urogenital Neoplasms	1.18E-08
	Collagen Diseases	1.59E-08
	Chorioamnionitis	2.04E-08
	Prostatic Neoplasms	2.65E-08
	Neoplastic Processes	7.08E-08
	Male Urogenital Diseases	8.39E-08
Cluster 7	Inflammation	1.68E-15
	Immune System Diseases	7.12E-15
	Chronic Disease	2.56E-11
	Common Cold	2.62E-11
	Respiratory Tract Infections	2.62E-11
	Autoimmune Diseases	3.01E-11
	Arthritis, Rheumatoid	4.31E-11
	Hypersensitivity	3.03E-10
	Infection	3.83E-10
	Joint Diseases	8.06E-10
Cluster 8	Neoplastic Processes	4.48E-36
	Neoplasm Metastasis	1.99E-31
	cancer or viral infections	5.98E-30
	Neoplasm Invasiveness	7.15E-29
	Carcinoma	1.44E-28
	Neoplasms	6.39E-28
	Fibrosis	4.27E-27
	Adhesion	8.04E-25
	Collagen Diseases	1.02E-24
	Adenocarcinoma	9.35E-23

Supplementary Table 6: The enriched miRNAs in the four clusters

See Supplementary File 3

Supplementary Table 7: The enriched transcription factor targets (TFT) in the four clusters

See Supplementary File 4

Supplementary Table 8: The genes in the nine sub-modules

M1	M2	M3	M4	M5	M6	M7	M8	M9
ACSM3	ADAM12	ALDH1A3	ACTG2	GALNT6	AMACR	DPP4	EFHD1	MXRA5
ANO7	CHAD	CA12	ARHGAP6	ST6GALNAC2	FOLH1	FAP		S100A16
CRABP2	COL11A1	CKB	CNN1			VCAN		FNDC1
CTHRC1	COL1A1	GDF15	GFRA1					TMPRSS2
FABP4	COL1A2	MAOA	MMP11					PGM5
FAM3B	COL3A1	PRUNE2	MYH11					PDE9A
GATA3	COL5A2	S100A14	SFRP2					TBC1D9
GPRC5A	COL6A3	SLC2A12						HGD
KLF15	DES	SPON2						MIR614
KRT19	ECM1	STC2						LRRC15
KRT7	FN1	STEAP1						STEAP2
MFAP2	PLAU	TRPS1						PCP4
MFAP5	POSTN							
NEFH	THBS2							
NKX3-1								
PLA1A								
SERPINA5								
SLC4A4								
TFAP2A								
TSPAN1								
WFDC1								

Supplementary Table 9: The used cancer types and number of the corresponding samples

Abbreviated cancer name	Tumor samples	Normal samples
BRCA	1119	113
KIRC	542	72
PRAD	502	52
THCA	513	59
LUAD	541	59
BLCA	414	19
COAD	483	41
STAD	420	37
UCEC	554	35
HNSC	504	44
READ	167	10
LIHC	374	50
